# Sex differences in the aging human heart: decreased sirtuins, pro-inflammatory shift and reduced anti-oxidative defense

**DOI:** 10.18632/aging.101881

**Published:** 2019-04-08

**Authors:** Maria Luisa Barcena de Arellano, Sofya Pozdniakova, Anja A. Kühl, Istvan Baczko, Yury Ladilov, Vera Regitz-Zagrosek

**Affiliations:** 1 Institute of Gender in Medicine and Center for Cardiovascular Research, Charité University Hospital, Berlin, Germany; 2 DZHK (German Centre for Cardiovascular Research), Berlin Partner Site, Berlin, Germany; 3 Charité – Universitätsmedizin Berlin, Corporate Member of Freie Universität Berlin, Humboldt-Universität zu Berlin, Berlin Institute of Health, iPATH.Berlin-Immunopathology for Experimental Models, Berlin, Germany; 4 Department of Pharmacology and Pharmacotherapy, Interdisciplinary Excellence Centre, University of Szeged, Szeged, Hungary

**Keywords:** cardiac aging, inflammatory response, sex differences, sirtuins, SOD2

## Abstract

Aging is associated with increased inflammation and alterations in mitochondrial biogenesis, which promote the development of cardiovascular diseases. Emerging evidence suggests a role for sirtuins, which are NAD^+^-dependent deacetylases, in the regulation of cardiovascular inflammation and mitochondrial biogenesis. Sirtuins are regulated by sex or sex hormones and are decreased during aging in animal models. We hypothesized that age-related alterations in cardiac Sirt1 and Sirt3 occur in the human heart and examined whether these changes are associated with a decrease in anti-oxidative defense, inflammatory state and mitochondrial biogenesis. Using human ventricular tissue from young (17-40 years old) and old (50-68 years old) individuals, we found significantly lower Sirt1 and Sirt3 expression in old female hearts than in young female hearts. Additionally, lower expression of the anti-oxidative protein SOD2 was observed in old female hearts than in young female hearts. Aging in female hearts was associated with a significant increase in the number of cardiac macrophages and pro-inflammatory cytokines, as well as NF-kB upregulation, indicating a pro-inflammatory shift. Aging-associated pathways in the male hearts were different, and no changes in Sirt1 and Sirt3 or cardiovascular inflammation were observed. In conclusion, the present study revealed a female sex-specific downregulation of Sirt1 and Sirt3 in aged hearts, as well as a decline in mitochondrial anti-oxidative defense and a pro-inflammatory shift in old female hearts but not in male hearts.

## Introduction

Aging, including cardiac aging, is a natural and multifactorial process characterized by a series of mechanisms, including deregulated autophagy, oxidative stress, systemic inflammation and mitochondrial dysfunction [1–9]. Recent studies have emphasized the importance of NAD^+^-dependent deacetylase sirtuins, mainly cytosolic- or nucleus-localized Sirt1 and mitochondrial-localized Sirt3, in the aging process [10]. Decreased sirtuin expression has been found in aged humans and animals [11,12]. Additionally, recent reports have suggested a significant reduction in the cellular NAD^+^ concentration and sirtuin activity in aged animals [11]. Furthermore, AMPK, a key regulator of cellular metabolism that significantly contributes to sirtuin activity, e.g., via increasing NAD^+^ synthesis [13], showed declined activity associated with aging [14].

The decline of Sirt1 and Sirt3 expression or activity may result in the following two detrimental consequences: first, it may impair mitochondrial biogenesis [15–17] and function [18,19], and second, it may lead to excessive inflammatory response, particularly due to the reduced anti-inflammatory actions of Sirt1 [20,21]. Several reports suggest that Sirt3 ameliorates mitochondrial stress by upregulating mitophagy and anti-oxidant machinery proteins, including manganese superoxide dismutase (SOD2) and catalase [22,23].

Both mitochondrial dysfunction and chronic inflammation are well-known hallmarks of aging [1,24,25]. Furthermore, in both cases, elevated ROS formation, another aging hallmark [26], is to be expected. Though the age-associated reduction in sirtuin expression and activity as well as its contribution to mitochondrial dysfunction and pro-inflammatory shift are well-documented, the underlying cellular mechanisms are far from understood.

Recent studies applying *in vivo* and *in vitro* models have suggested a role for the female sex hormone estradiol (E2) in the expression and activity of AMPK and sirtuins [27,28]. Apart from being the downstream target of AMPK, Sirt1 deacetylates the upstream activator of AMPK, LKB1 kinase, thus providing the positive feedback loop between AMPK and Sirt1 [29,30]. Of note, E2 blood concentrations are reduced in aged women, i.e., after menopause. In fact, postmenopausal may women have lower E2 concentrations in their blood compared with age-matched men [31]. The loss of E2 is accompanied by the release of pro-inflammatory cytokines, leading to activation of inflammatory pathways in aging [32]. Furthermore, loss of ovarian hormones due to reproductive aging leads to the decline in anti-oxidative defense, mitochondrial biogenesis and function in females [33,34].

Though several reports have demonstrated sex differences in the expression of AMPK and sirtuins in mouse brain and kidney [35], alterations in the human heart in aging remain unknown. Therefore, in the present study, we aimed to investigate the age-related alterations in Sirt1, AMPK and Sirt3 signaling in cardiac tissue from men and women along with markers for mitochondrial biogenesis, anti-oxidative defense and the inflammatory state. We found a female sex-specific downregulation of Sirt1 and Sirt3 expression in aged hearts, which is accompanied by the downregulation of SOD2, a key mitochondrial anti-oxidative enzyme, and by increased expression of inflammatory mediators.

## RESULTS

### Age-related Sirt1 and AMPK alterations in women

**Figure 1:**
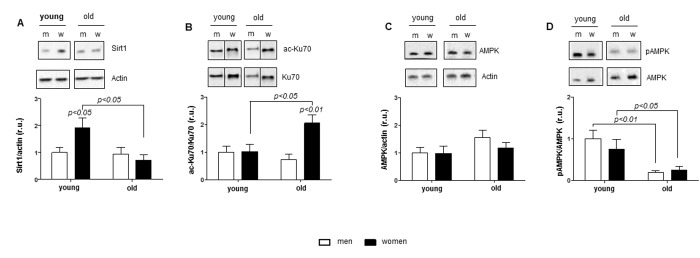
**Age-related alterations in Sirt1 and AMPK expression.** Western blot expression analysis of (**A**) Sirt1, (**B**) acetylated Ku70, (**C**) total AMPK and (**D**) phosphorylated AMPK (Thr172) performed with human cardiac tissue lysates from young and old men (m) or women (w). pAMPK was normalized to AMPK. Proteins were normalized to actin. Data are shown as the mean ± SEM (n= 6-8/group). Representative imaging of western blot analysis; the lanes were run on the same gel. All data were normalized to the corresponding control and expressed in relative units (r.u.).

The expression of Sirt1 and AMPK is downregulated in aging [11]; however, sex differences in this process remain unknown. To examine this issue, we analyzed the expression and activity of Sirt1 and AMPK in human cardiac tissue. Sirt1 expression was significantly reduced in old compared to young females (p< 0.05) (Figure 1A). To test whether the Sirt1 downregulation is associated with altered protein acetylation, acetylation of the nuclear protein Ku70, a direct target of Sirt1, was analyzed. Consistently, Ku70 acetylation in aged female hearts was significantly elevated compared to young women (p< 0.05) (Figure 1B). In contrast, neither Sirt1 expression nor Ku70 acetylation were altered in male hearts with aging.

Analysis of AMPK expression and the phosphorylation rate, i.e., pAMPK/AMPK, revealed a significant decrease in AMPK phosphorylation with aging in both sexes (Figure 1D) in the presence of unchanged total AMPK contents (Figure 1C).

### Age-related expression of mitochondrial and anti-oxidative enzymes in women and men

**Figure 2:**
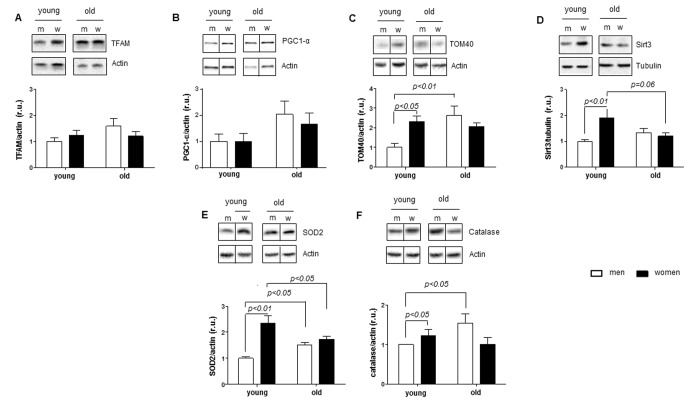
**Age-related alterations in expression of mitochondrial and anti-oxidative enzymes.** Expression of proteins related to mitochondrial function in non-diseased hearts in young and old men (m) and women (w). Western blot analysis and statistical analysis of (**A**) TFAM, (**B**) PGC1-α, (**C**) TOM40, (**D**) Sirt3, (**E**) SOD2 and (**F**) catalase protein expression. Proteins were normalized to tubulin or actin. Data are shown as the mean ± SEM (n= 6-9/group). Representative imaging of western blot analysis; the lanes were run on the same gel. All data were normalized to the corresponding control and expressed in relative units (r.u.).

A key role has been suggested for Sirt1 and AMPK in the regulation of mitochondrial biogenesis and function [13,37–39]. In the next step, we tested whether the female sex-specific downregulation of Sirt1 in aged female hearts is accompanied by an alteration in mitochondrial biogenesis markers. By analyzing the expression of two key transcription factors involved in mitochondrial biogenesis, i.e., mitochondrial TFAM and nuclear PGC1-α, we found no age- or sex-related alterations (Figure 2A-B). Interestingly, expression of TOM40, a key protein controlling mitochondrial protein import, was markedly upregulated in aged male hearts (Figure 2C). In agreement, no sex- or age-related differences were found in the expression of mitochondria-encoded (*cox1* and *nd4*) or nuclear-encoded (*atp5b*, *cox5b* and *nsufs1*) mitochondrial genes (Supplementary Figure 1).

In contrast, analysis of the expression of two key mitochondrial proteins that control the acetylation of mitochondrial proteins (Sirt3) and the mitochondrial redox state (SOD2) revealed their significantly lower levels in old female vs. young female hearts (Figure 2D-E). Similar to SOD2, the expression of catalase, a key peroxisomal and mitochondrial anti-oxidative enzyme, was significantly lower in old compared to young female hearts (Figure 2F).

In contrast to females, expression of SOD2 and catalase was significantly elevated in male hearts. Thus, heart aging is associated with a reduction in anti-oxidative defense in women (SOD2), whereas this defense is increased (SOD2 and catalase) in male hearts (Figure 2E-F).

### Aged-related pro-inflammatory state in women

An enhanced systemic pro-inflammatory state is a characteristic feature of aging [40]. Thus, we aimed to examine whether alterations in Sirt1 expression and anti-oxidative defense may be accompanied by pro-inflammatory reactions in aged female hearts.

**Figure 3:**
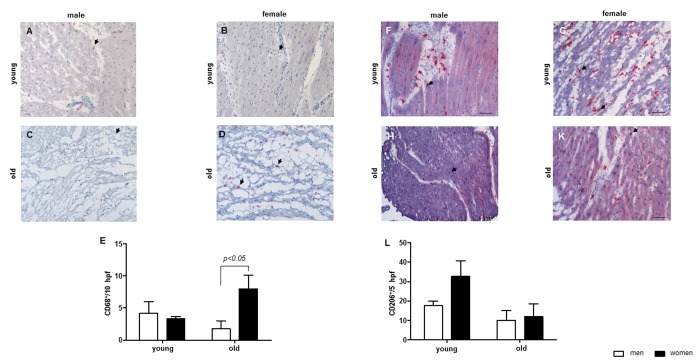
**Aging effect on the number of macrophages in cardiac tissue.** Representative images of cardiac cryosections stained with antibodies against (**A**-**D**) CD68, followed by (**E**) statistical analysis of CD68-positive cells per high power field (hpf). (**F**-**K**) CD206, followed by (**L**) statistical analysis of CD206-positive cells per high power field (hpf). The analyses were performed with myocard from young and old men (m) or women (w). Data are shown as the mean ± SEM (n= 6-8/group). Arrows show CD68- or CD206-positive cells.

By analyzing the number of cardiac macrophages, a significant increase in the cardiac macrophage number was observed in old female hearts, but not in male hearts (Figure 3A-E). The amount of CD206-immune reactive macrophages was higher in young women when compared to old women or to young men (Figure 3F-L).

**Figure 4:**
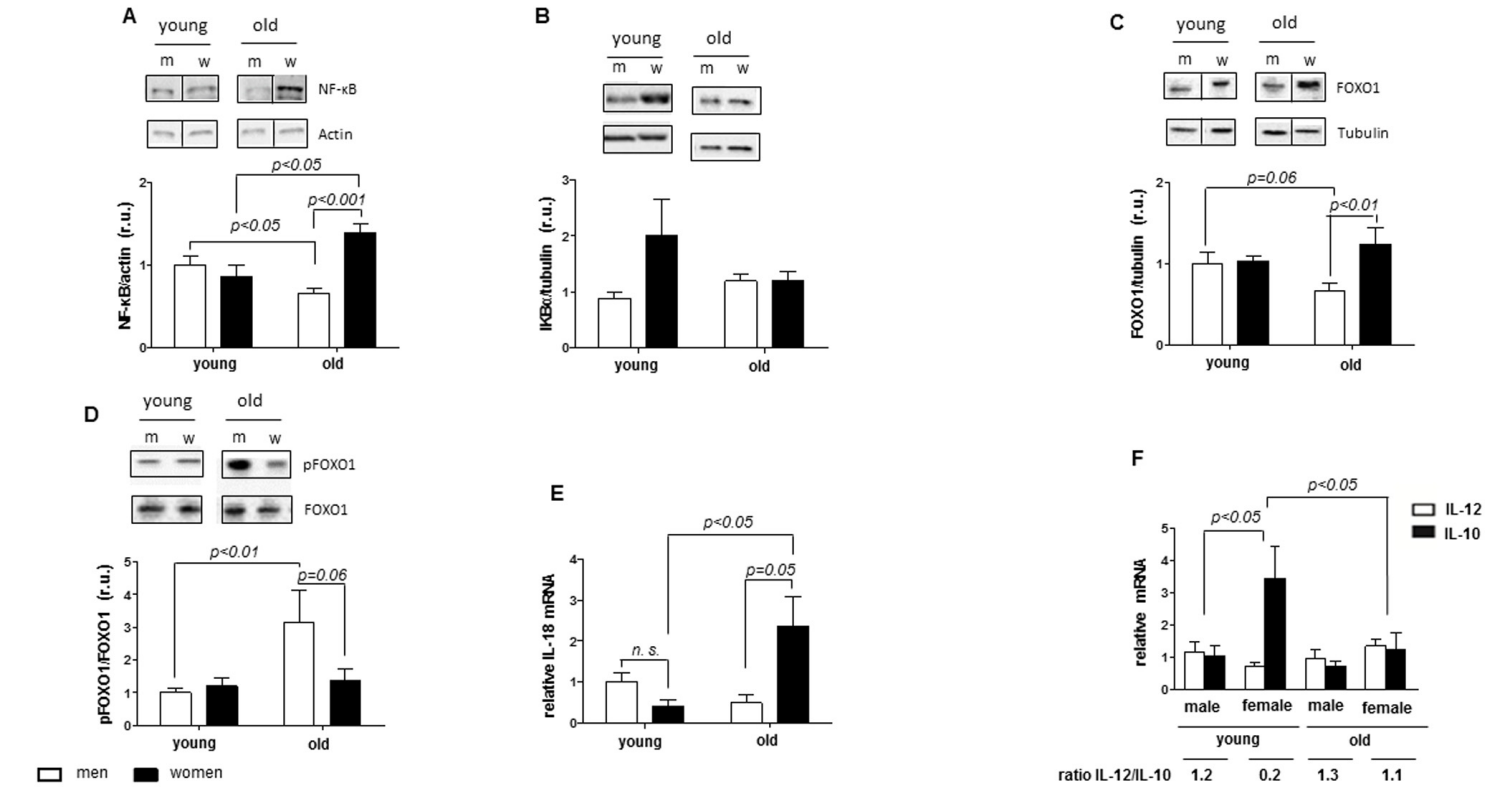
**Female-specific aged-induced pro-inflammatory state.** Protein and relative mRNA expression of pro-inflammatory and anti-inflammatory mediators in non-diseased hearts in young and old men (m) and women (w). Western blot analysis of (**A**) NF-κB p50, (**B**) FOXO1 and (**C**) phosphorylated FOXO1 and real-time PCR analysis of (**D**) IL-18, (**E**) IL-12 and (**F**) IL-10 mRNA expression. Data are shown as the mean ± SEM (n= 6-9/group). pFOXO1 was normalized to total FOXO1. Representative imaging of western blot analysis; the lanes were run on the same gel. All data were normalized to the corresponding control and expressed in relative units (r.u.).

To support this finding, expression analysis of pro- and anti-inflammatory factors was performed. NF-κB p50 was significantly elevated in aged female hearts, whereas it was markedly reduced in aged male hearts (Figure 4A). In contrast, IKBα was reduced in aged female hearts when compared to young female hearts (Figure 4B).

It has been suggested that FOXO1 phosphorylation suppresses inflammatory responses via NF-κB [41]. FOXO1 inactivation increases with aging in male animals [41], suggesting that an increase in FOXO1 phosphorylation might contribute to the reduced level of inflammation in old animals.

FOXO1 was decreased in old male individuals when compared to young male hearts (p= 0.06), but not in female hearts (p< 0.05) (Figure 4C). FOXO1 phosphorylation was altered in cardiac male tissue during aging (p< 0.01), while unchanged in female hearts during aging (Figure 4D). The phosphorylation of FOXO1 (p> 0.05), was similar in female and male cardiac tissues in young individuals (Figure 4D).

Similar to NF-kB, the mRNA of pro-inflammatory cytokine IL-18 was upregulated in old female hearts but not in male hearts (Figure 4E). The TNF mRNA was unchanged in male and females hearts during aging (p> 0.05) (data not shown).

Further analysis of the IL12/IL10 ratio revealed a significantly lower ratio in young female hearts (ratio= 0.2, p< 0.05) compared to young male hearts (1.2). This sex difference was lost in aged hearts, i.e., 1.1 in old female hearts and 1.3 in old male hearts, which also confirmed the pro-inflammatory shift in aged female hearts (Figure 4F).

## DISCUSSION

The aim of the study was to examine age-related changes in Sirt1, AMPK and Sirt3 expression in women and men in relation to mitochondrial biogenesis, anti-oxidative defense and inflammation in human hearts. The main findings are as follows: (i) Aging leads to a significant downregulation in Sirt1 expression and the corresponding elevated acetylation of nuclear proteins in female but not male hearts; (ii) The higher expression of some mitochondrial and anti-oxidative proteins in young females is lost with aging either due to their downregulation in aged females, e.g., Sirt3 and SOD2, or due to upregulation in aged males (TOM40, SOD2 and catalase); and (iii) Aging leads to a significant pro-inflammatory shift in female but not male hearts.

AMPK and Sirt1 are partner proteins orchestrating a wide variety of intracellular processes including cellular resistance to oxidative stress, general metabolism, inflammation, and mitochondrial biogenesis and function [42]. Aging is accompanied by the downregulation of AMPK and Sirt1 activity in animals and humans [43]. Whether these alterations are sex-dependent remains unknown. We found a sex-independent downregulation of the AMPK phosphorylation rate, a widely used indicator of AMPK activity. Furthermore, the female-specific downregulation of Sirt1 expression was observed in aged hearts. Consistently, acetylation of Ku70, a nuclear protein and a direct Sirt1 client, was significantly increased only in old female but not male hearts, demonstrating reduced Sirt1 activity. Although we did not investigate whether the nuclear or cytosolic Sirt1 content is specifically reduced with aging in female hearts, previous reports demonstrated that Sirt1 physically interacts with and deacetylates nuclear Ku70 [44].

Mitochondrial dysfunction is a characteristic fingerprint of aging [45]. Notably, downregulation of Sirt1 activity has been previously attributed to disturbances in mitochondrial biogenesis [19]. To examine the age-related effects on mitochondrial biogenesis in male and female hearts, expression analysis of key transcription factors (PGC1alpha and TFAM) as well as several nuclear- and mitochondria-encoded mitochondrial proteins was performed by western blot and PCR. Surprisingly, we found no sex- or age-related differences in the expression of mitochondrial genes or PGC1alpha and TFAM expression, which excludes the sex-dependent regulation of mitochondrial biogenesis in aging human hearts, even though such changes were reported in lower organisms or in the liver and kidneys from 22-month-old mice. Species and organ differences may account for these discrepancies [35,46].

Previous reports argue for an activating effect of Sirt1-dependent deacetylation on PGC1alpha [47,48]. It is tempting to speculate that female-specific Sirt1 downregulation and enhanced acetylation of nuclear proteins (e.g., Ku70) may also be accompanied by PGC1alpha hyperacetylation and inactivation. Indeed, considering that PGC1alpha directly interacts with the SOD2 promoter regions and regulates its expression [49], the observed downregulation of SOD2 in aged female but not male hearts argues for a potential reduction in PGC1alpha activity.

Several studies have demonstrated favorable expression of anti-oxidative enzymes in female vs. male hearts, which is lost in postmenopausal women [50]. In agreement with these findings, the expression of two key anti-oxidative enzymes, i.e., SOD2 and catalase, in our study was significantly higher in young female vs. male hearts, whereas this sex difference is lost in aged hearts. Interestingly, this age-related alteration in the sex difference in the anti-oxidative enzymes is due to their opposite regulation in female and male hearts. Indeed, SOD2 expression in aged female hearts was downregulated, whereas expression of both enzymes was upregulated in aged male hearts. This finding leads to an intriguing idea that there are mechanisms activated in aged male hearts leading to improved anti-oxidative defense. Though the underlying cellular mechanisms upregulating the anti-oxidative enzymes SOD2 and catalase in aged male hearts still have to be elucidated, previous reports demonstrated an elevation in blood estrogen concentrations in males with age [31]. Given that estrogen upregulates anti-oxidative enzymes including SOD2 [51,52], one may assume that the known age-related changes in the estrogen concentration in blood from males (increase) and females (decrease) may contribute to the sex difference in SOD2 and catalase expression observed in our study.

Similar to SOD2, a sex-dependent alteration in the expression of Sirt3, a major regulator of the mitochondrial acetylome, was observed in our study. Global analysis of the mitochondrial protein acetylome performed by Herbert et al. [53] revealed a several-fold increase in the acetylation of multiple lysine residues in the liver of Sirt3-deficient mice. The net contribution of Sirt3 activity comprises the regulation of mitochondrial dynamics [54] and function [55], i.e., OXPHOS activity, ATP synthesis and fatty acid oxidation. Therefore, downregulation of Sirt3 expression in aged female hearts may lead to increased mitochondrial protein acetylation of i.e. SOD2 and disturbed mitochondrial functions, which may further exacerbate the reduced anti-oxidative defense in the mitochondria, i.e., reduced SOD2 expression.

Aside from the transcriptional regulation of Sirt3 expression, a recent study by Kwon et al. [12] suggested an unexpected mechanism for the post-translational regulation of Sirt3 activity and stability via Sirt1-mediated deacetylation. Indeed, the authors found the presence of Sirt1 in the mitochondria and its interaction with Sirt3. The Sirt1-driven deacetylation of Sirt3 significantly increases its stability and enzymatic activity. Of note, the authors observed a hyperacetylation, and therefore instability of Sirt3 in aged mice. Based on our finding, i.e., reduced Sirt1 and Sirt3 expression in aged female hearts, one may suppose a female-specific downregulation of this novel Sirt1-Sirt3 axis. Altogether, the current study provides convincing evidence of female sex-specific downregulation of Sirt1 and Sirt3 accompanied by disturbed expression in some mitochondrial proteins and anti-oxidative defense.

In addition to regulating mitochondrial function and biogenesis, emerging data also suggest the role of Sirt1 in acute and chronic inflammatory response, e.g., via inhibition of transcription factor NF-kB [20,21]. Since, chronic inflammation is a characteristic feature of aging [40,56], we were wondering whether female sex-specific Sirt1 downregulation may be accompanied by enhanced inflammation in aging female hearts. Indeed, the present study revealed a significant pro-inflammatory shift in aged female but not male hearts demonstrated by an increase in the cardiac macrophage content as well as upregulation of NF-kB, IL-12 and IL-18 expression, specifically in hearts from old women. In contrast, the anti-inflammatory protection was significantly decreased in women with age, which was confirmed by the elevation of the IL-12/IL-10 ratio. Similar increase in the IL-12/IL-10 ratio in the brain of old female mice has been found by Zhang et al. [57]. Interestingly, it seems that NF-κB is only involved in the inflammatory response in female but not involved in male hearts based on the data of NF- κB, IKBα and the pro-inflammatory cytokines.

In agreement with pro-inflammatory shift in heart of aged women, an increased number of CD68-positive macrophages along with increased pro-inflammatory cytokine expression was found. However, the macrophages found in the cardiac tissue from young women seem to be anti-inflammatory macrophages, since these macrophages were CD206 positive. A shift in the IL-12/IL-10 ratio to the site of IL-10 might indicate that the cardiac macrophages in the cardiac tissue of young women are regulatory macrophages, since regulatory macrophages produce high amounts of IL-10 and low amounts of IL-12 []^64^]. This shift is lost in aged women. Altogether, the findings from this study argue for a female sex-specific pro-inflammatory shift in aged hearts. Although the underlying mechanisms of this shift remain unclear, the female sex-specific downregulation of Sirt1 and SOD2 expression suggest a potential contribution.

Sexual hormones modulate the immune system via hormone receptors and regulate inflammation [58]. Although both the pro- and anti-inflammatory actions of estrogens have been described [59], the majority of studies argue for the anti-inflammatory effects of estrogen receptor activation. Particularly, E2 exhibits anti-inflammatory actions on endothelial and immune cells *in vitro* [60,61]. E2 loss leads to the expression of pro-inflammatory cytokines in humans [32]. In human activated peripheral blood mononuclear cells (PBMCs), E2 inhibits the expression of pro-inflammatory cytokines [62] and decreases NF-κB activity [63].

One limitation of the study is that our cohorts are relatively small and the human material archived high heterogeneity. Due to the small sample number, the statistical significance might not accurately show age or sex differences.

In conclusion, the present study revealed a female sex-specific downregulation of Sirt1 and Sirt3 in aged human hearts accompanied by a decline in the mitochondrial anti-oxidative defense and a pro-inflammatory shift.

## MATERIALS AND METHODS

### Human left ventricular samples

Human non-diseased whole lateral left ventricular (LV) wall tissue was collected from organ donors (men= 16 and women= 15). The whole tissue was frozen immediately after collection in liquid nitrogen and stored at -80°C. The donors were between 17 and 68 years of age. We divided the LV samples into 4 groups: young (17-40 years; male: n= 7 and female: n= 7) and old (50-68 years; male: n= 9 and female: n=8) individuals.

We obtained the informed consent from all donors or their legal guardians. Sample collection and the experimental protocols were approved by the Scientific Board at the Hungarian Ministry of Health (ETT-TUKEB: 4991-0/2010-1018EKU). All research was performed in accordance with the German and Hungarian guidelines.

### RNA extraction and quantitative real-time PCR

Total RNA isolation from cardiac human tissue as well as quantitative real-time PCR were performed as previously described [36]. The mRNA contents of target genes were normalized to the expression of ribosomal protein large P0 (RPLP0) or hypoxanthine phosphoribosyl transferase (HPRT). The purity of the isolated RNA was analyzed with the Bioanalyzer “caliper LabChip” (Agilent Technologies, Rattingen, Germany).

### Protein extraction and immunoblotting

LV samples were homogenized in RIPA buffer (50 mmol/l Tris-HCl, pH 7.4, 150 mmol/l NaCl, 1 mmol/l EDTA, 1% NP-40, and 0.25% Na-deoxycholate) supplemented with protease inhibitor cocktail (Roche, Mannheim, Germany), and the phosphatase inhibitors sodium orthovanadate (1 mmol/l Na_3_VO_4_) and sodium fluoride (1 mmol/l NaF). Proteins were quantified using the BCA Assay (Thermo Scientific Pierce Protein Biology, Schwerte, Germany). Equal amounts of total proteins were separated on SDS-polyacrylamide gels and transferred to a nitrocellulose membrane. The membranes were immunoblotted overnight with the following primary antibodies: Sirt3 (1:1000, Cell Signaling, USA), Ku70 (1:200, Santa Cruz, USA), ac-Ku70 (1:2000, Lys 331, Abcam, UK), TFAM (1:200, Santa Cruz, USA), SOD2 (1:1000, Santa Cruz, USA), catalase (1:1000, Cell Signaling, USA), Sirt1 (1:1000, Cell Signaling, USA), AMPK (1:2000, Cell Signaling, USA), p-AMPK (1:2000, Thr172, Cell Signaling, USA), FOXO1 (1:1000, Cell Signaling, USA), p-FOXO1 (1:1000, Ser 256, Cell Signaling (USA), TOM40 (1:1000, Abcam, UK) and NFκB p50 (1:1000, Santa Cruz, USA). Equal sample loading was confirmed by analysis of actin (1:1000, Santa Cruz, USA) and tubulin (1:50.000, Sigma-Aldrich, USA). Immunoreactive proteins were detected using ECL Plus (GE Healthcare, Buckinghamshire, UK) and quantified with ImageLab (version 5.2.1 build 11, Bio-Rad Laboratories (USA)).

### Immunohistochemistry

For immunohistochemistry, 5 µm cryo-sections of human left ventricle were fixed in formalin for 1 hour at room temperature and subjected to a heat-induced epitope retrieval step prior to incubation with anti-CD68 antibody (clone PGM-1, Agilent Technologies, Santa Clara, CA, USA). The detection was performed by the LSAB method applying the Dako REAL™ Detection System (Agilent Technologies, Santa Clara, CA, USA). Nuclei were counterstained with hematoxylin and mounted on slides with glycerol gelatin (both Merck KGaA, Darmstadt, Germany). Negative controls were performed by omitting the primary antibody. Images were acquired using an AxioImager Z1 microscope (Carl Zeiss MicroImaging, Inc.). Positive cells were quantified in 5 high power fields (hpf) (field of vision in x400 original magnification). All evaluations were performed in a blinded manner.

### Statistical analysis

The data are given as the mean ± SEM. The GraphPad Prism 5 (GraphPad Software, 2003, San Diego, USA) was used for the statistical analysis. The data were evaluated using the non-parametric test (Mann-Whitney test, for two independent groups) or two-way analysis of variance (to test two independent variables). The Bonferroni post-test was used as a post hoc test. Statistical significance was accepted when p < 0.05**.**

## Supplementary Material

Supplementary File
