# Supplementary material for: Sex differences in the aging human heart: decreased sirtuins, pro-inflammatory shift and reduced anti-oxidative defense
Source: Aging (Albany NY). 2019 Apr 8;11(7):1918–33. doi: 10.18632/aging.101881 (PMC6503880; doi:10.18632/aging.101881)

SUPPLEMENTARY MATERIAL

Supplementary Figure

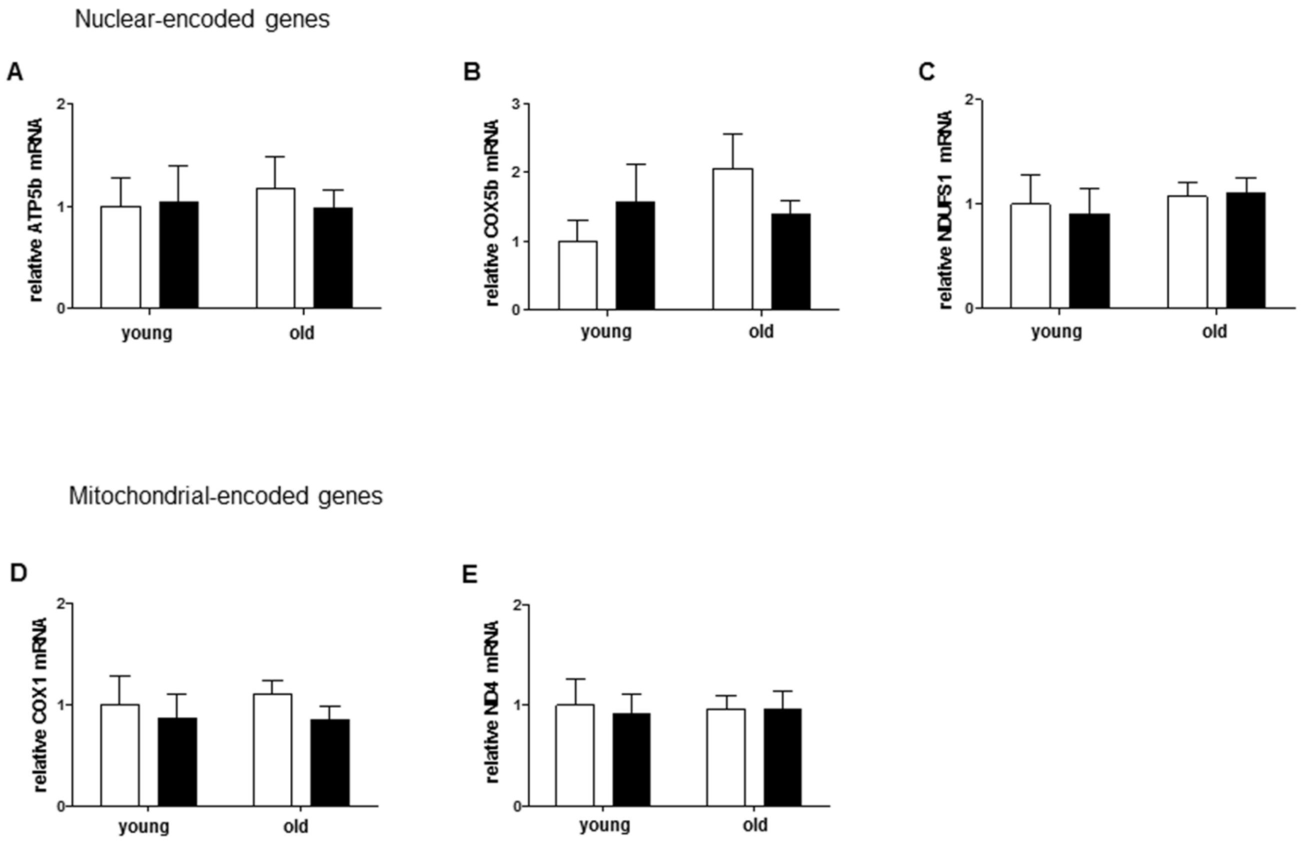

Supplementary Figure 1. No sex- or age-dependent differences in mitochondrial biogenesis. Relative mRNA expression of nuclear- and mitochondrial-encoded genes in non-diseased hearts in young and old men and women. Real-time analysis for the nuclear-encoded genes (**A**) ATP5b, (**B**) COX5b and (**C**) NDUFS1 and mitochondrial-encoded genes (**D**) COX1 and (**E**) ND4. Data are shown as the mean ± SEM (n= 6-9/group).

Original Blots

Original Blot 1.

1	2	3	4	5	6	7	8	9	10	11	12	13	14	15	16	17	18	19	20	21	22	23	24	25	26	27	28	29	30	31	32	33	34	35
M	1945	1650	1940	1941	1942	1944	1946	1961	1964	1957	1958	1959	1960	1963	1947	1949	1950	1951	1952	1953	1954	1955	1956	1790	1968	1969	1970	1971	1972	1973	M	M	M	

WB 07.06.16

20µg / lane; 6%; nitrocellulose membrane

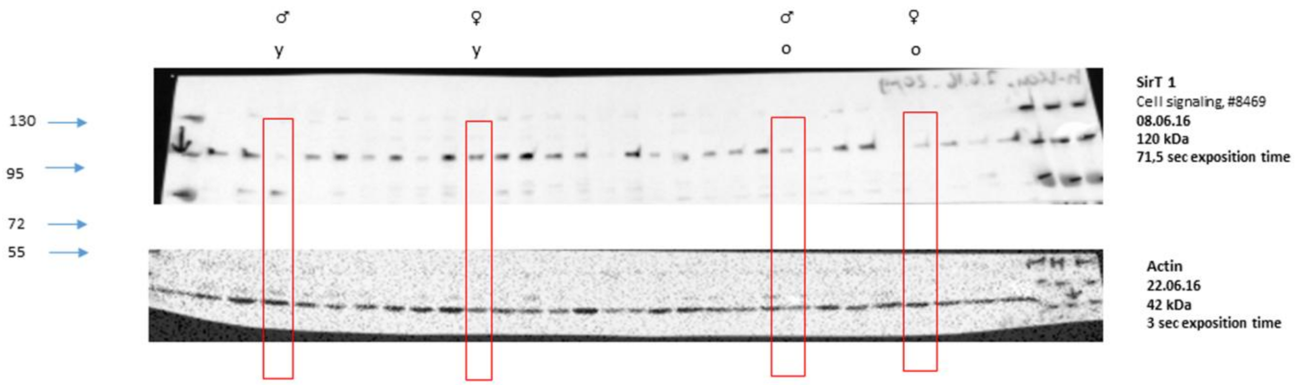

Original Blot 2.

1	2	3	4	5	6	7	8	9	10	11	12	13	14	15	16	17	18	19	20	21	22	23	24	25	26	27	28	29	30	31	32	33	34	35
M	1945	1650	1940	1941	1942	1944	1946	1961	1964	1957	1958	1959	1960	1963	1947	1949	1950	1951	1952	1953	1954	1955	1956	1790	1968	1969	1970	1971	1972	1973	M	M		

WB 18.02.16

15µg / lane; 10%; nitocellulose membrane

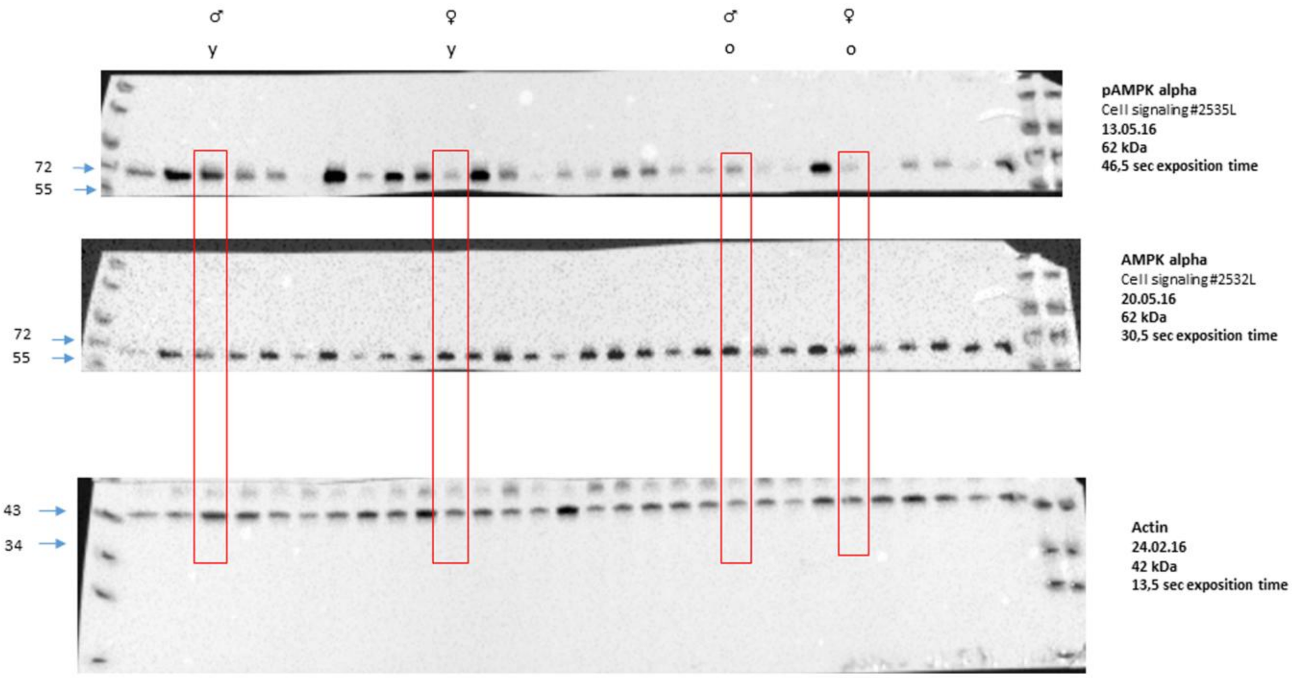

Original Blot 3.

1	2	3	4	5	6	7	8	9	10	11	12	13	14	15	16	17	18	19	20	21	22	23	24	25	26	27	28	29	30	31	32	33	34	35
M	1945	1650	1940	1941	1942	1944	1946	1961	1964	1957	1958	1959	1960	1963	1947	1949	1950	1951	1952	1953	1954	1955	1956	1790	1968	1969	1970	1971	1972	1973	M	M		

WB 18.02.16

15µg / lane; 10%; nitocellulose membrane

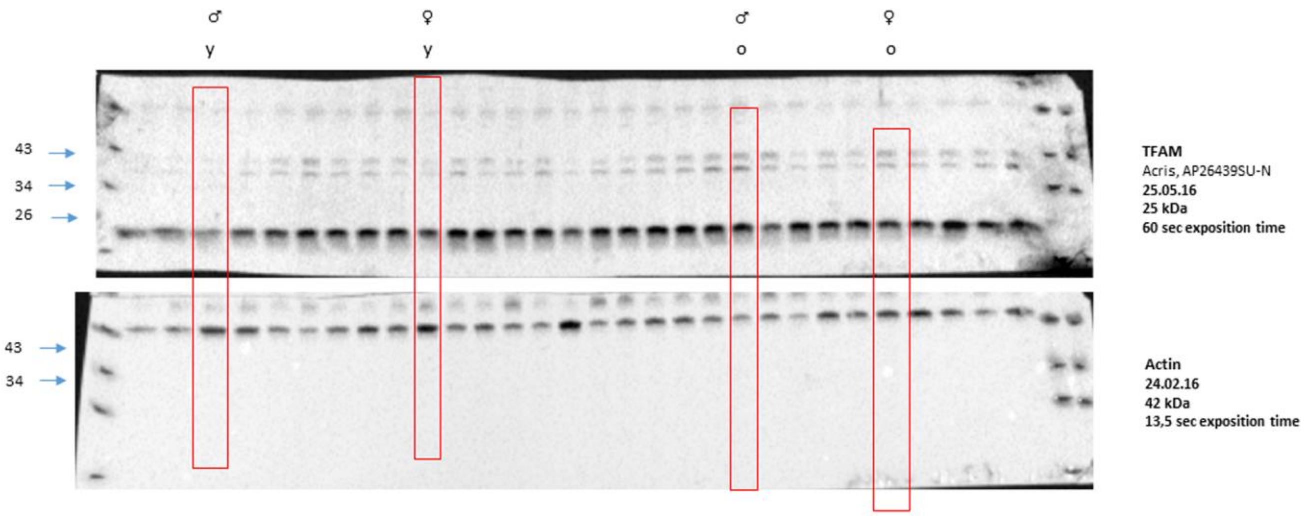

Original Blot 4.

1	2	3	4	5	6	7	8	9	10	11	12	13	14	15	16	17	18	19	20	21	22	23	24	25	26	27	28	29	30	31	32	33
M	1945	1650	1940	1941	1942	1944	1946	1961	1964	1957	1958	1959	1960	1963	1947	1949	1950	1951	1952	1953	1954	1955	1956	1790	1968	1969	1970	1971	1972	1973	M	M

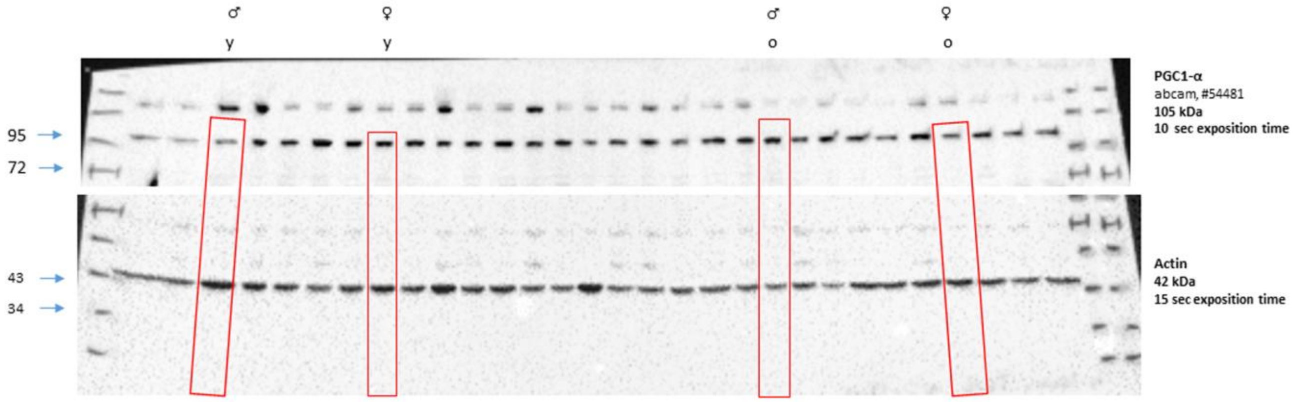

Original Blot 5.

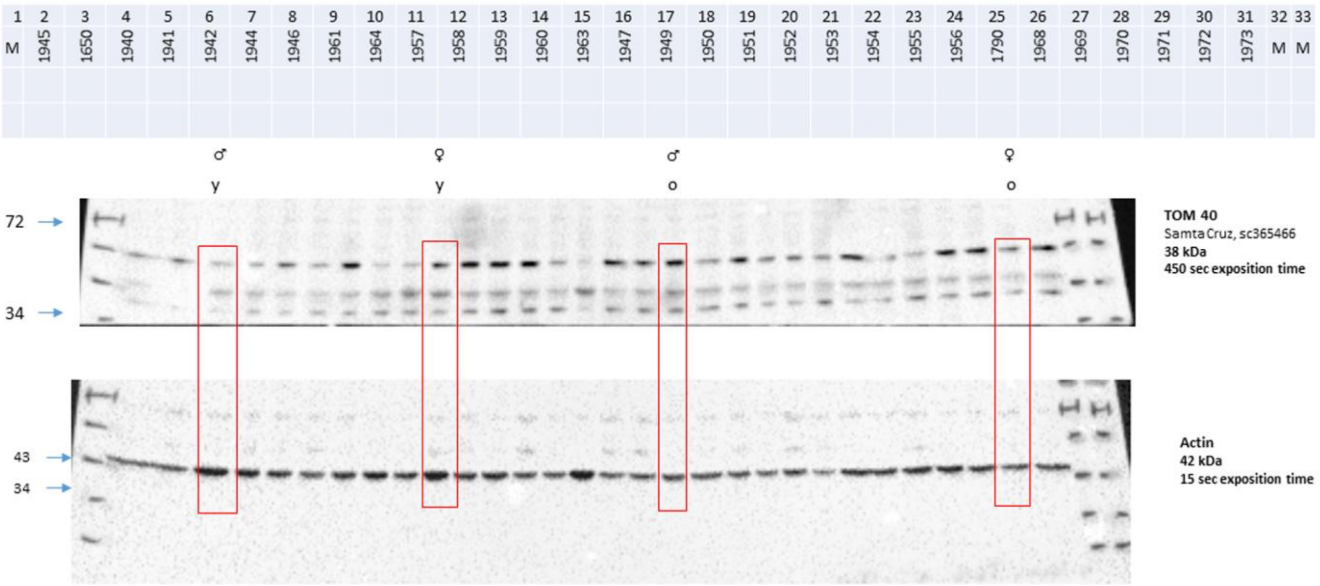

Original Blot 6.

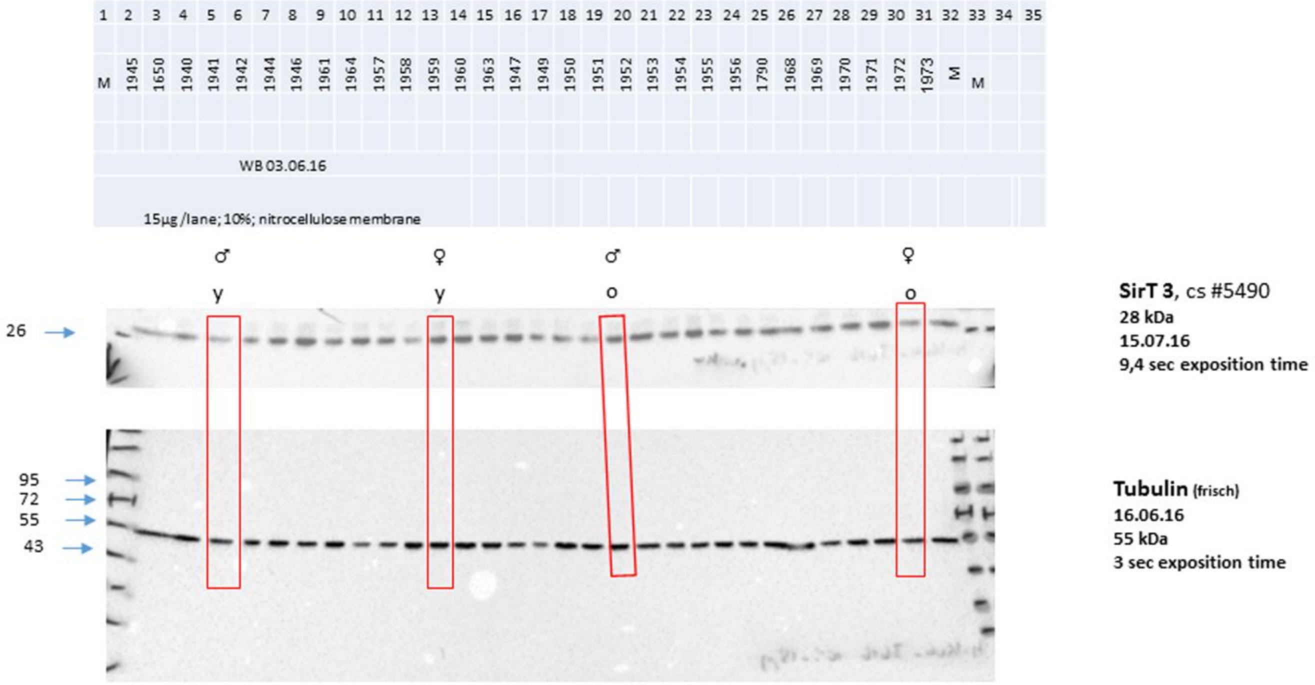

Original Blot 7.

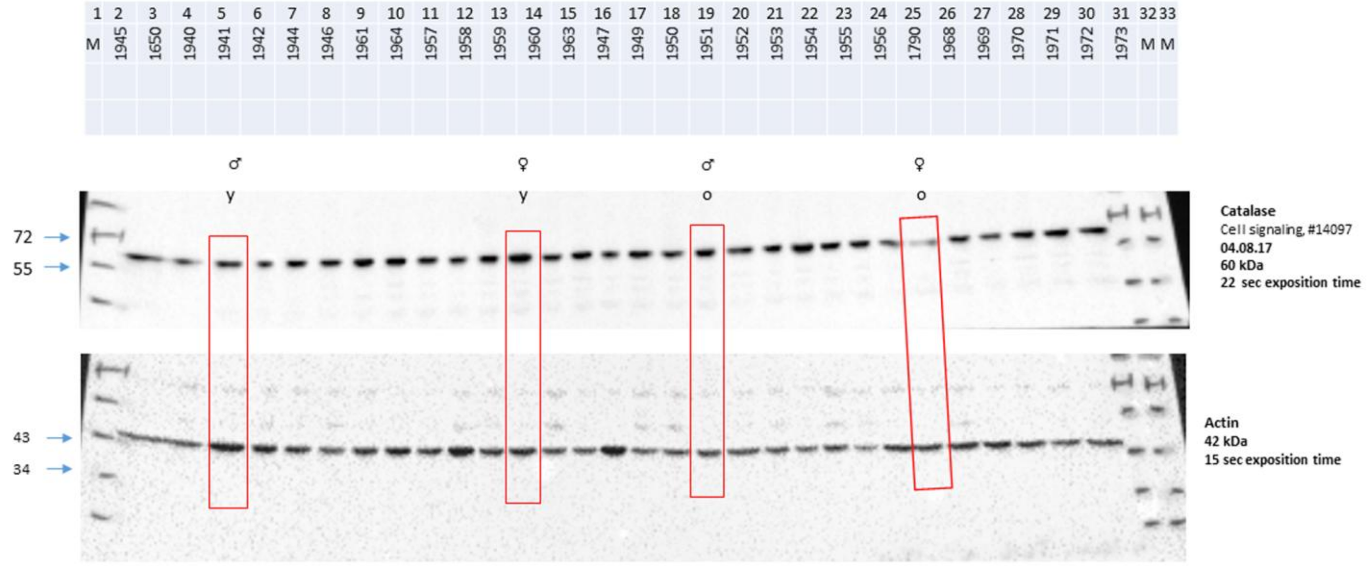

Original Blot 8.

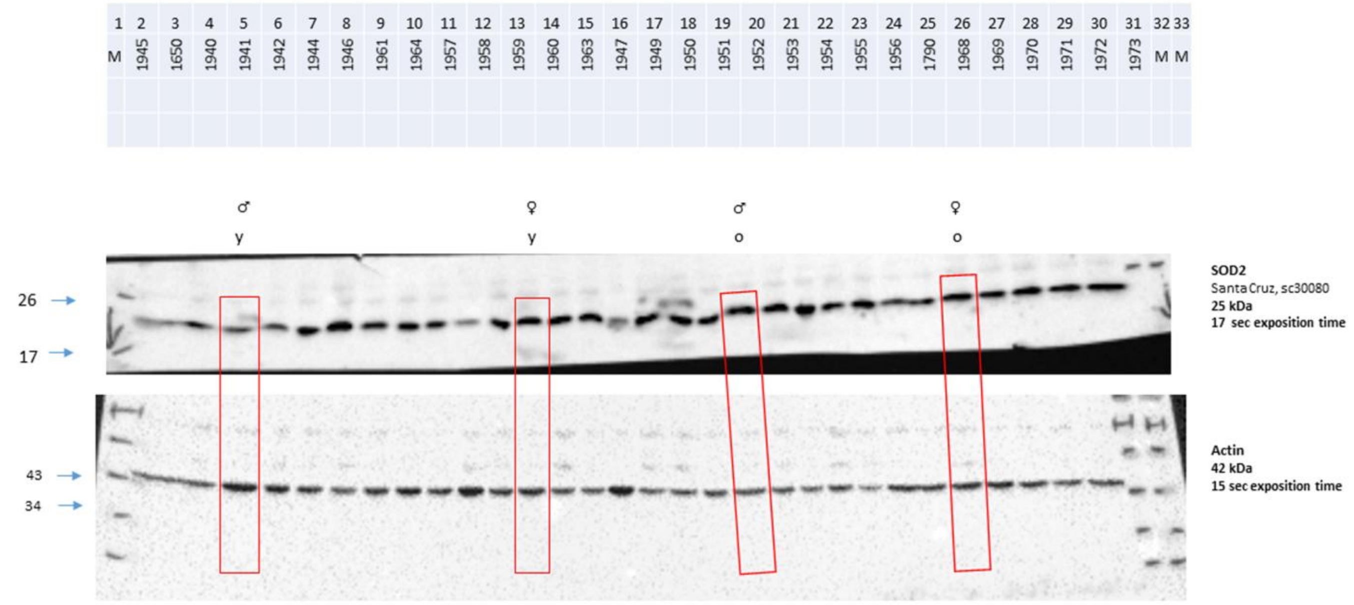

Supplement: Supplementary File [file aging-11-101881-s001.pdf]
